# Dingo movement depends on sex, social status and litter size

**DOI:** 10.1098/rsos.250255

**Published:** 2025-07-30

**Authors:** Brendan F. Alting, Benjamin J. Pitcher, Michelle Campbell-Ward, Neil R. Jordan

**Affiliations:** ^1^ Centre for Ecosystem Science, School of Biological, Earth and Environmental Sciences, University of New South Wales, Sydney, New South Wales, Australia; ^2^ Taronga Conservation Society Australia, Mosman, New South Wales, Australia; ^3^ School of Natural Sciences, Faculty of Science and Engineering, Macquarie University, Sydney, New South Wales, Australia; ^4^ Sydney School of Veterinary Science, Faculty of Science, The University of Sydney, Camden, New South Wales, Australia

**Keywords:** canid, territoriality, camera trapping, reproduction, dingo, seasonal behaviour

## Abstract

Territoriality constrains animal movement as resident individuals or social groups defend areas from non-residents. Here, we evaluated space use by dingoes, a territorial and socially monogamous group-living apex predator in Australia. We used data from remote camera traps and hourly fixes from GPS collars on eight individuals in five packs to identify variations in dingo territoriality and movement leading up to and including their annual breeding season, particularly in relation to an individual’s known social status, sex and competition within their pack. Subdominant male detections increased outside their pack’s home range during the breeding season, while subdominant female detections were unchanged. Furthermore, dominants spent more time (a higher proportion of detections) inside their territory as the number of pups present in their pack from the previous year increased. In common with other carnivores, these results suggest that ranging patterns depend on the sex and breeding status of the individual and potentially on levels of competition. Subdominant males may be exploring breeding opportunities outside of their own range, while dominants may remain in their territory to defend space, resources and reproductive partners. Understanding individual movement within and beyond their home range, can help to guide management actions both spatially and temporally.

## 1. Introduction

Territoriality is the defence from conspecifics of spatially defined resources (e.g. food [1]; den or shelter sites [2]; offspring [3] or mates [4]) and is found across the taxonomic spectrum including in birds (e.g. white-bellied antbird, *Myrmezica longipes* [5]), mammals (e.g. voles and lemmings [6]), fish (e.g. zebrafish, *Danio rerio* [7]) and reptiles (e.g. lace monitors, *Varanus varius* [8]). For territory holders, the potential costs of physical conflict arising from efforts to exclude conspecific competitors should be outweighed by the benefits of primary access to these resources [9]. However, the value of resources varies over time and so investment in territoriality may shift depending on resource pulses [10] or annual breeding cycles when defending mates becomes more critical [11]. Determining how territoriality varies seasonally, and uncovering the mechanisms behind these changes, is critical for understanding species biology and the functions of territorial behaviour, with potential implications for wildlife management [12].

Many canid species are socially monogamous and territorial, and several live in social groups consisting of a dominant breeding pair, supported by non-breeding offspring from previous litters [13]. Most canid species have high reproductive skew, with breeding monopolized by the dominant pair, although under certain circumstances multiple females reproduce (e.g. African wild dogs, *Lycaon pictus* [14]). Breeding typically occurs annually in dingoes [15] (variously referred to as *Canis dingo*, *Canis familiaris*, *Canis lupus dingo*, among others [16]), as in other wild canids [4]. In the denning/pup rearing period, pups are constrained to the vicinity of a den or lair before gradually becoming more mobile [4], and home range size and movement dynamics can therefore change considerably during this period [17]. In contrast, much less focus has been given to shifts in the pre-breeding and breeding periods and how this might reflect investments in territoriality and resource or mate defence more broadly.

The function of territoriality in canids has been discussed in the literature [18,19], with the role of resource acquisition clearly important. When territories are held year-round, their size is usually determined by the minimal amount of space required to sustain residents throughout the most resource-limited period of the year [20]. Territoriality may also play a role in the acquisition and defence of other resources, such as mates, particularly for dominant/breeding individuals. Mixed paternity occurs in overtly socially monogamous species, such as African wild dogs [14] and dingoes [21], while extra-territorial males sire more than a quarter of offspring in Ethiopian wolves (*Canis simensis* [22]) and Arctic foxes (*Vulpes lagopus* [23]). Resident breeding males are subject to increased territorial incursions by reproductive competitors during the breeding season (e.g. Ethiopian wolves [22]) and use various strategies to retain dominance and ensure paternity, including mate-guarding (grey wolves, *Canis lupus* [24]; bat-eared fox, *Otocyon megalotis* [25]), establishing seasonal territories (red foxes, *Vulpes vulpes* [26]), or focusing territorial behaviours such as scent marking at this time (coyotes, *Canis latrans* [27]). In contrast, breeding females may benefit from extra-pair copulations as confusing the paternity of the litter may reduce infanticide risk [28], and guard against the risk of paired male infertility [4]. Dominant females must balance these potential benefits of extra-territorial incursions by males against the need to defend against takeover by rival females [4].

The contrasting strategies of dominant males and females, and dominants and subdominants, may lead to different patterns of territory use during the breeding season. In canids, dominant individuals generally produce the bulk of the group’s offspring and may have more incentive to adhere to their territorial structures than subdominant individuals. On the other hand, subdominant individuals that are members of a pack generally do not receive direct reproductive benefits of maintaining this territory, and in some species, dispersers reproduce more than ‘biders’ who stay in a territory [29]. Remaining within the territory, however, can have indirect fitness benefits [4]: it is generally safer than dispersing, and temporary forays from the territory may be a way of trading off temporary danger with the chance to reproduce, while also gaining information on surrounding packs [30]. When an influx of new pups is born into the pack, and competition for food or reproductive opportunities increases, the benefits of dispersing may outweigh the benefits of biding, despite potential indirect fitness benefits to subdominants of helping raise related offspring, particularly for males [31]. For example, in wolves, high wolf density is associated with subdominant dispersal [32]. Resource competition within the pack will also affect dominants, but the direct fitness benefits of siring young outweighs the increase in competition for food. We thus may expect to see contrasting patterns of territory use between dominant and subdominant individuals, potentially dependent on factors such as resource competition and breeding opportunities.

Dingoes are a medium-sized canid present in varying densities across the vast majority of mainland Australia [33]. Dingoes are highly adaptable, occurring in most Australian ecosystems, and can alter their diet and sociality depending on food availability [34]. Similar to other canid species, dingo packs consist of a dominant/breeding pair, and sometimes offspring from previous years. As in other canids such as coyotes and black-backed jackals (*Lupulella mesomelas* [35]), dingo social structure is flexible, depending on resource availability [34]. For instance, high resource availability within the natal range may encourage offspring to stay rather than disperse [36], resulting in a larger pack size, while in areas with less resources smaller groups may be more common [37]. Dingo packs/pairs are territorial, with neighbouring home ranges overlapping minimally, if at all [15]. Packs exhibit fission–fusion dynamics, such that members of a pack will sometimes forage or commute alone or in sub-groups, and then later reunite [38]. Offspring may disperse from their natal packs as early as the age of 1 year, but sometimes remain within the pack for multiple years, with packs consisting of between approximately 2 and 10 individuals [39]. Dingoes in a pack will respond aggressively to individuals intruding into their territory [15], and residents scent mark throughout their territory, potentially advertising territory ownership [40]. As such, dingoes are a suitable model species for studying variations in breeding behaviour between dominant and subdominant individuals, particularly in the context of known territories.

Dingoes have distinct breeding phases throughout the year. The breeding season generally occurs between mid-February and May, with some studies showing behaviours such as scent marking in males heightened at this time [15]. Within this period, females are reproductively receptive for approximately two weeks, during which they may breed with multiple males [15]. Gestation is approximately nine weeks, and females are mostly restricted to the den site and its vicinity for a further two−three months (approx. May–September) following parturition [39]. Pups begin to emerge from the den in August/September, but are not fully mobile until November/December.

To investigate dingo ranging behaviour, we used a combination of camera traps and direct observations to identify individual dingoes and their social status. Using GPS collar data from eight individuals from five contiguous packs of wild dingoes in the period leading up to and including the annual breeding season, we compared detections of dingoes within and outside their territorial range. We predicted that:

(a) Overall, detections of dingoes would increase towards the end of the pre-breeding season as occurs in other seasonally breeding species (e.g. African wild dogs [17]).
(b) During the pre-breeding period, dominant dingoes would be detected more frequently within their pack’s home range, due to the potential heightened need to defend their territory from intruders [41], while subdominants would be detected more outside of their pack’s home range to explore extra-territorial breeding opportunities [42]. In particular, we expected subdominant male dingoes to be observed more out of their territories, as that would increase the chances of breeding with an unrelated dominant female.
(c) If male mate-guarding of breeding females occurs during the breeding season, then lone detections of dominant females would decrease during the breeding period, relative to the three-month period leading up to this [43].
(d) The proportion of pre-breeding season movements in different territory zones would change dependent on the number of pups that remain in the pack from the previous year’s litter. Specifically, while parents (dominants) may remain in close proximity to their core range as pups begin to leave the den, subdominant individuals may be expected to increase exploration and dispersal, due to a possible increase in resource competition [44], resulting in greater detections outside of their pack’s home range. As such, breeding females may be expected to visit the peripheries of their home ranges during the pre-breeding/breeding season, to increase the likelihood of contact with additional unrelated males.

## 2. Methods

### 2.1. Study site

**Figure 1.**
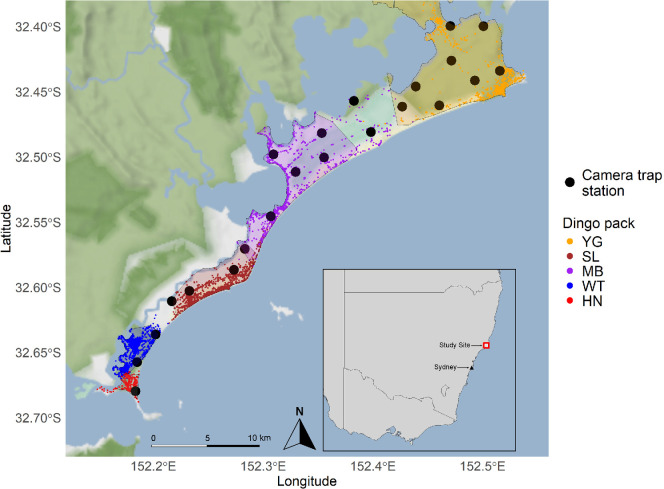
Raw GPS data points representative of five dingo packs in the mid-coast region of NSW Australia (June 2021–November 2021), with 95% minimum convex polygon (MCPs) overlaid. Camera traps (black dots) were active from November 2021 to May 2022.

This study was conducted on Worimi Country in the Great Lakes region (centred on 32.492° S, 152.343° E) of the mid-coast of eastern Australia, NSW (figure 1). The area is composed of several land tenures, including national parks, council land, private land and urban and peri-urban areas. It is a popular recreational and tourist destination, with activities such as fishing, cycling and camping occurring throughout the area and year. The area is composed of a mosaic of different habitats, including extensive coastal sand dunes, woodland (dominated by *Angophora costata* and *Banksia serrata*), paperbark swamps (dominated by *Melaleuca quinquenervia*), coastal wetlands and littoral rainforests. Dingoes are common [45] and visible throughout the area, being frequently seen throughout the town of Hawks Nest in the south, and in and around campgrounds within the national park.

### 2.2. GPS collar data

To allow researchers to fit GPS radio-collars to representative dingoes from each pack, seven dingoes were live trapped between April 2021 and April 2023 using large walk-in cage traps baited with mullet or chicken necks. One additional dingo was captured using a Victor soft catch foot trap with padded jaws. Dingoes were anaesthetized by an experienced wildlife veterinarian with an intramuscular injection of a combination of medetomidine (0.04−0.06 mg kg^−1^), ketamine (2.9−4.6 mg kg^−1^) ± butorphanol (0.1 mg kg^−1^) administered through a blow dart, and later reversed with an intramuscular injection of atipamezole (5× the medetomidine dose). Collars were fitted, and morphometric data were recorded while dingoes were anaesthetized (less than 1 h). For this study, seven individuals were fitted with GPS collars (Advanced Telemetry Systems W500), with data downloaded through an ultra high frequency (UHF) link during monthly welfare checks. One further individual was collared with a satellite collar (Advanced Telemetry Systems G5), which transmitted data through satellite once daily. Collars weighed between 286 and 357 g, less than 5% of adult dingo body weight (mean trapped dingo weight = 15.7 kg, range = 13.1–18.7 kg). GPS collars recorded hourly positions, and remain attached at the time of writing (*n* = 2), were removed when the animal was next trapped (*n* = 1), subsequently dropped off through a cotton release (*n* = 2), or were collected when the animal died (*n* = 3). All research was conducted under University of New South Wales Animal Ethics Committee approvals (19/119B and 22/102A) and all research dingoes were released under Scientific licences (SL102302 and SL102716).

### 2.3. Camera traps

Twenty-two camera stations with paired cameras (*n* = 44 cameras, Reconyx hyperfire H2x1000) were deployed for six months, from December 2021 to June 2022, and again for six months between December 2022 and June 2023 (figure 1). One camera trap was stolen in the second year, and for consistency data from this camera site in the first year were excluded. Cameras in high human use areas were secured with security boxes and locked to trees using python locks. Cameras stations were placed on average 2380 m apart, and faced inwards, on each side of a trail, road, or 4wd track (hereafter ‘trail’) to capture both dingo flanks [45]. Cameras were mounted to trees approximately 0.5 m from the ground, pointed slightly angled to trails [46]. This two-camera set-up reduced the likelihood of dingoes being present at a site but passing behind the detection zone. Cameras were set to record images in bursts of three, in rapid fire, with medium sensitivity. Batteries and SD cards were changed every 36 days (range = 28–45 days). Cameras generally were placed in the same location in both years, but were moved to a nearby location if vegetation growth made locations unsuitable, or if cameras were stolen from a given site, during the study.

### 2.4. Image processing

Camera trap images were analysed using the open-source AI machine learning program Megadetector, which separates images into detections of humans, animals, vehicles or empty [47]. The minimum confidence threshold was set at 15% confidence for animals using the program ‘Timelapse’ [48] and images with less than 15% confidence were removed from the dataset. One researcher manually identified images to the species level in the remaining subset of images. Dingoes were identified to the individual level separately by two researchers. A dingo identity profile was created each time a new individual was detected on the cameras, consisting of photos of the left and right flank of the dingo. Dingoes were identified using their unique sock patterns, which differ in shape and extent, as well as scars and coat colour [45]. Researchers then compared their identifications and discussed any differences in opinion, which were all resolved by consensus.

### 2.5. Dingo classifications

We used counts of independent dingo detection events (camera detections greater than 30 min apart for each individual) as our response variable, noting this variable is dependent on dingo population density and camera trap location [49]. We restricted our dataset to detections of known, individually identified individuals from the two-camera trap survey sessions. Given that detection probability at a given site is influenced by animal movement behaviours [50], using counts of independent detections as a proxy of increased movement is appropriate if comparing detections from the same array of camera stations across time. Given the large number of independent dingo detections (*n* = 1135) and individuals identified (*n* = 34) across both survey years, changes in independent dingo detections at camera stations are probably representative of changing dingo movement. We included only adult dingoes in our movement analysis, categorizing all as either dominant or subdominant. Dominant individuals were defined as pairs that had been consistently seen together, observed tandem scent marking, mating or bearing pups (for females) [27,51], either from direct observations or from camera trap video footage. Adult individuals (individuals over 1 year old) that were not part of a dominant pair, but were part of a pack, were considered subdominant. Transient individuals (i.e. not associated with any pack) were discarded from further analysis. Transient individuals were classified as those which were identified regularly traversing multiple territories, a behaviour that would be unlikely for resident individuals. This dingo population has been under study since 2019, such that pack dynamics, the identity of dominant individuals and the age, origin and subdominant status of other individuals are known from direct observations, and are generally stable year on year, or any fluctuations are known and documented.

We used pups that we identified from camera traps that were observed travelling with the dominant pair as our ‘pups in pack’ metric. While pup attrition to 1 year may be high, the number of pups identified during the survey (approximately six months after their birth) is a reasonable indicator of potential current and near-future resource competition in the natal territory.

### 2.6. Dingo territorial borders

Where possible, we used four months of collar data from each individual leading up to the start of the camera trap survey (December in both sessions), to best approximate a baseline home range area for each pack, based on individuals collared within these packs. We excluded GPS fixes with horizontal dilution of precision (hdop) greater than 2 [52] and visually inspected the GPS data to exclude any clearly erroneous points, such as those in adjacent lakes or the ocean. We then subset each dataset to 2000 random points, to account for autocorrelation of fixes in the datasets [53]. We identified home range zones for each individual by defining each individual’s home range as the 95% minimum convex polygon (MCP) estimate, a well-established method of home range approximation [54]. MCPs provide a less reliable estimate of home range size compared with other estimators, but are useful for studying approximate territorial boundaries between individuals [55], particularly for a geographically closed study site such as ours. The collared individuals in this area had non-overlapping home ranges, and ranges are contiguous, covering the majority of the study area (figure 1).

We then assigned each camera station as falling either within or outside of each individual dingo’s ‘pack’ home range. Only two cameras changed status across the two sessions—one changed from ‘outside home range’ to ‘inside home range’ for one pack, and one changed from ‘inside home range’ to ‘outside home range’ for the same pack (electronic supplementary material S1). When an identifiable dingo was detected at a camera trap we classified it as being detected either inside or outside of its pack’s territory. If dingoes were detected that we could not assign to a pack (e.g. transient individuals that were not in our identification catalogue), we excluded them from further analysis, as all camera stations would be defined as extra-territorial for these individuals, inflating the number of extra-territorial detections.

### 2.7. Data analysis

To test predictions (a), (b) and (c), we fitted generalized additive models (GAMs) in the *mgcv* package in R, designed specifically for analysing time series data [56]. We fitted one model to test hypotheses (a) and (b), with counts of dingo detections per ‘week’ (7 day periods) as a continuous response variable. The selection of week-long intervals was somewhat arbitrary, but was selected to achieve a balance between the animal having time to traverse its entire home range and being short enough to capture temporal variation. For each ‘week’ with no detections, we included this as a count of 0. We included a random effect of ‘session’ to account for variation between camera survey years, and a random effect of ‘pack’ to account for variation in pack home range size and behaviour (*n* = 5 packs in year session). We then fitted a spline term (bs = ‘cr’) to the ‘week’ variable, to identify variation in total counts leading up to and including the breeding season (hypothesis (a)). We also added another spline with the ‘week variable, but included an interaction term (StatusSexZone), combined of ‘status’ (dominant or subdominant individual), ‘sex’ (male or female) and ‘zone’ (whether the detection was inside or outside the individuals pack home range), to identify if the combination these factors (eight levels) influenced counts of detections by week (hypothesis (b)). We also added this interaction term as a stand-alone term (table 1) to see if counts differed overall between groups. For hypothesis (c), we reduced our dataset to only dominant individuals, and split the remaining detections into three categories: paired detections (male and female detected at same camera less than 5 min apart), males detected alone and females detected alone. We fitted another model with count as a response variable, including an interaction between type of detection (lone male, lone female or paired) and zone (within territory, outside territory), and again also included this as a stand-alone term in the model, along with the random effects of ‘pack’ and ‘session’.

**Table 1.** List of model terms for each. Models run in the ‘mgcv’ package [56] and ‘lme4’ [57].

prediction	model	response variable	model formula	family
(a) and (b): *dingo movement based on status, sex and home range zone*	GAM	detections, all individuals	~s(week, k = 7, bs = ‘cr’)+s(week, by = StatusSexZone, k = 7, bs=‘cr’)+StatusSexZone + s(Session, bs = ‘re’)+s(Pack, bs = ‘re’)	negative binomial
(c): *dominant pair/single detections*	GAM	detections, dominant individuals	~s(week, k = 7, bs = ‘cr’)+s(week, by = TypeZone, k = 7, bs = ‘cr’)+TypeZone + s(ession, bs=‘re’)+s(Pack, bs ‘re’)	negative binomial
(d): *dependent offspring affect movement of dominants*	GLMM	ratio of detections in home range to detections out of home range	~pups in pack*StatusSex + (1|Session) +{1|Pack)	binomial

We fitted both GAMs with a negative binomial distribution, to account for overdispersion in the data [58]. We assessed significance of model terms on detection rates by evaluating the coefficient estimates and associated *p*-values. We assessed the significance of any smooth terms in the model by observing the predicted model plots and assessing the estimated degrees of freedom (which describes the knots in the spline), along with associated *p*-values.

For hypothesis (d), we fit a generalized linear mixed model (GLMM) in the *lme4* package [57] in R, with counts of each individual dingo in different territorial zones across the entire study period as a response variable and a binomial distribution. We used the number of ‘pups in pack’ from the previous year (which ranged between zero and eight) with an interaction with ‘status’ (dominant or subdominant) and ‘sex’ (male or female) as independent variables. Time measured in months was not included in this model as we were interested in ratios of visits within and outside pack home ranges dependent on pups in the previous year, not the timing of these events. We again included ‘session’ and ‘pack’ as random effect terms in the model.

For all three models, the small number of variables tested and plausibility of all predictors in explaining the response variable justifies the use of the full models to assess significance of these variables [59].

## 3. Results

### 3.1. GPS collaring

We collected GPS collar data from eight individuals, representative of 10 identified dingo packs in the study area across 2 years (electronic supplementary material, table S1). This consisted of five packs in each year: JB pack, WT pack, SL pack, MB pack and YG pack. The study area is relatively constrained, and as such, we are confident this data covers the majority of the area (figure 1). Of the eight collared dingoes, four were dominant and four were subdominant (electronic supplementary material, table S1). Given the stability of pack ranges across survey years, it is unlikely that the dominance status of collared dingoes will affect their pack’s territorial ranges. For two of the packs, a lack of a collared individual in one year made it necessary to use the same collar dataset for both years. For one individual, we had reliable collar data from only April to October, and these data were used to estimate the territory boundary for this pack in both years. Sightings data from all packs in the area indicate that the ranges were the same in both years for these periods, and that pack home ranges are relatively stable over multiple years, particularly in the context of cameras classified as within or beyond home range boundaries. Territories of each of the five packs in both years contained an average of 3.6 cameras per territory (range = 1–6 cameras). Collared individuals and GPS dates used for analysis can be found in electronic supplementary material, table S1.

### 3.2. Summary results of camera trap data

A total of 854 187 images were captured across all survey periods. After *megadetector* processing, 160 486 ‘animal’ images remained (although due to the low confidence threshold we set, some ‘false positive’ images remained that did not contain an animal image). In total, 42 469 images contained one or more dingoes. This consisted of 1135 independent detection events of individually identifiable adult dingoes across the survey period, and 49 unidentifiable daytime images. In total, 34 adult dingoes were identified across the two six-month camera-trapping sessions, with some individuals identified in both years ($\bar{\text{x}}$ = 21.5 individual adults identified each year; s.d. ± 2.7). These individuals were members of five packs in the first session, and six packs in the second session. We discarded five individuals from further analysis as we could not assign them to a pack (i.e. transient individuals) and the space use and behaviour of these individuals will differ from territorial dingoes. We also removed another pair for which we had no GPS collar data. This left five known packs in total for both years for which we had GPS collar data (figure 1).

In each session, 10 dominant dingoes were detected from camera traps. In session one, nine subdominant dingoes were identified, and in session two, eight subdominants were identified. We identified 27 pups across the two years: 14 in the first year, and 13 in the second year. Of the adult individuals identified and included in analyses in the second year, eight had been identified as pups in the previous year. In session 1, each dominant individual was detected on average 44 times across the session (range = 21–75 detections), while subdominant individuals were detected on average 24 times (range 4−62 detections). In session 2, dominants were detected on average 36 times (range = 7–64 detections) and subdominants on average 14 (1–49 detections). From 9 of 10 packs (across the two sessions), dominant pairs were captured on camera together on average 24 times in each session (range = 14–45 sightings), enough to give confidence in assigning them as a dominant pair. One pair (JB pack, session 2) were not seen together on the cameras, but were seen consistently by researchers in the field, and on other cameras deployed that were not part of this survey.

### 3.3. Dingo movement based on status, sex and home range zone

**Figure 2.**
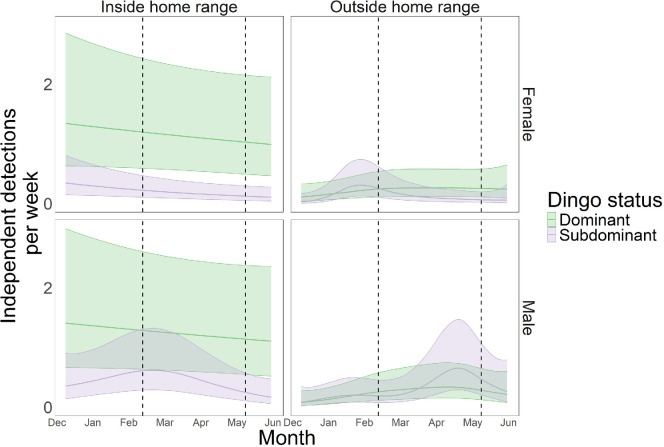
GAM conditional effects of individual dingo detections per week either inside or outside their home range, from two six-month camera trap surveys on the mid-coast of NSW, Australia, December 2021 to June 2022, and December 2022 to June 2023. The pre-breeding period occurs from December to February, while the breeding period occurs from March to May, and dashed line represents approximate start and end of breeding season. Raw data points presented in electronic supplementary material, S3. Figures and analysis used mgcv, ggplot and tidygam in R [56,60,61].

We found no evidence to support hypothesis (a); that overall dingo movement increased leading up to and during the breeding period (estimated degrees of freedom of spline term (edf) = 1.000, *p* = 0.683). Dingo detections per week varied depending on the pack of the dingo (edf = 3.917, *p* < 0.001), while session had a small effect on detection rate (edf = 0.947, *p* < 0.001). Overall, detections varied by group, with dominants detected more inside their home range than dominants outside their home range, subdominants inside their home range, or subdominants outside their home range (electronic supplementary material, table S2a; figure 2). We found some evidence to support hypothesis (b), as subdominant male detections outside the home range varied with time (edf = 3.954, *p* = 0.022) (figure 2), with detections increasing during the breeding season. Subdominant male dingoes also showed a slight increase in detections inside their home range at the start of the breeding season (edf = 2.471, *p* = 0.055), which was not predicted. Subdominant females showed an increase in detections outside their home range just before the breeding season (edf = 2.647, *p* = 0.027), although this effect was small. We found no evidence that detections of any other category of dingo varied with time, either inside or outside the home range (electronic supplementary material, table S2b). For brevity, full model results and significance of smooth terms are presented in supplementary materials (electronic supplementary material, tables S2a and S2b).

### 3.4. Dominant pair/single detections

**Figure 3.**
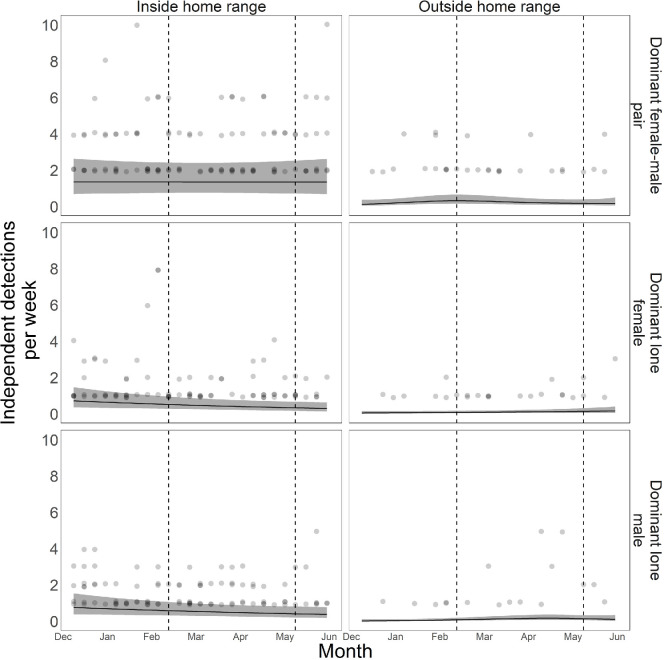
GAM predicted camera trap detections of lone and paired dominant dingoes, dependent on month, sex and their home range zone. Dots are raw data points, with darker circles representing more observations for that value per month with zeroes removed for clarity. Dashed line represents approximate start and end of breeding season. Figures and analysis used mgcv, ggplot and tidygam in R [56,60,61].

Overall detections varied between groups, with detections of paired dominants per week being highest within their home range (electronic supplementary material, table S3a; figure 3). We found limited evidence for hypothesis (c), with none of the dominant paired visits or lone dominant male visits varying with week, either inside or outside the home range (electronic supplementary material, table S3b; figure 3), while lone dominant female detections declined slightly inside the home range during the breeding season (edf = 1.000, *p* = 0.035), although this effect was minimal. ‘Pack’ had a significant effect on detection rates for dominant individuals (edf = 3.813, *p* < 0.001). ‘Session’ had no effect on dominant detection rates (edf = 0.656, *p* = 0.092). Females were rarely detected outside of their home range, particularly during the breeding season (figure 3).

### 3.5. Dependent offspring affect movement of dominants

**Figure 4.**
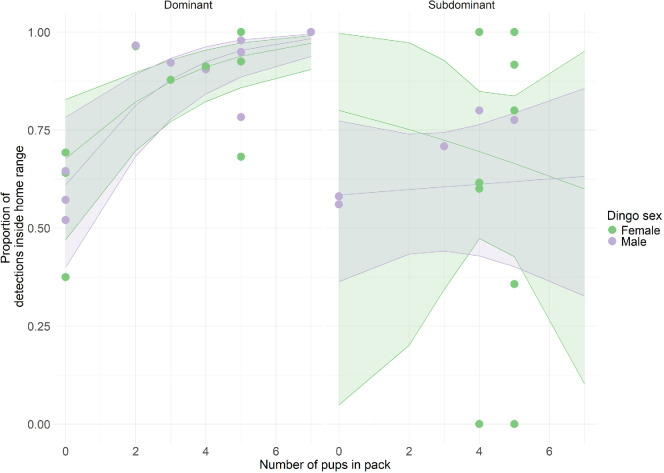
Proportion of camera detections of dingo individuals within their pack’s home range (dots), and GLMM model predicted fixed effects. Data from two six-month camera trap surveys from the mid-coast of NSW, Australia from December 2021 to May 2022, and December 2022 to May2023. ‘Number of pups in pack’ refers to the number of pups present in each pack the preceding year. Figures and models used lme4, ggeffects and ggplot2 [57,60,62].

Session as a random effect had no influence on the model for hypothesis (d), resulting in a singular fit, and was removed from further analysis. The number of pups in the pack from the previous year had no overall effect on dingo detection rates inside the home range relative to outside the home range (estimate = 0.029, s.e. = 0.126, *p* = 0.816). We found strong evidence of an interaction between the number of pups present and dingo status affecting the proportion of detections, both for dominant females (estimate = 0.373, s.e. = 0.098, *p* < 0.001), and dominant males (estimate = 0.492, s.e. = 102, *p* < 0.001) (figure 4), with dominant individuals more likely to spend time inside the home range with an increasing number of pups in their pack. Subdominant dingoes showed no increase or decrease in detections within the home range depending on the number of pups in the pack (figure 4).

## 4. Discussion

For purportedly monogamous animals with distinct breeding seasons, individual movement changes during and preceding this time can shed light on the function(s) of territoriality [4]. Animal movement can be affected by resource availability, intraspecific competition and individual social status [63]. Here, we show that dingo movement—when considered at the population scale—did not vary between the pre-breeding and breeding periods. However, differences were apparent when an individual’s sex and dominance status were considered. Specifically, subdominant male dingoes exhibited more extra-territorial movements leading up to and during the breeding season compared with at other times. In contrast, dominants spent more time inside their pack’s home range as the number of pups in their natal pack increased, while the number of pups had no impact on subdominant ranging. Dominant dingoes were mostly detected in pairs within their home range, and female dominants were very rarely seen alone outside their home range, particularly during the breeding season.

### 4.1. Dingo movement based on status, sex and home range zone

Many territorial species display biased movement patterns conditional on their breeding status, including black-backed jackals [64]. In territorial species, extra-territorial forays probably yield greater rewards for subdominants during the breeding season through mating opportunities (hypothesis (b)). Here, we found that subdominant male dingoes increased their movement during the breeding period. They also showed a slight increase in detections within their home range in February, immediately before the breeding season, perhaps suggesting some exploratory behaviours during this time (figure 2). This mirrors data from meerkats (*Suricata suricatta*), where subdominant males increase intrusions during the breeding season [65]. For males that successfully reproduce ‘sneakily’ with resident females in adjacent territories, this is a low-cost investment as the ‘sneaker’ male subsequently does not contribute to raising the young [66]. Similarly, in estuarine crocodiles (*Crocodylus porosus*), subdominant males move substantially more during the breeding season than dominant males, purportedly to find new available territories [67]. Similarly, our results could indicate that subdominant male dingoes are prospecting for vacant territories or pursuing a ‘sneaker’ strategy by attempting to breed with resident females in adjacent territories, as occurs in bank voles (*Clethrionomys glareolus* [68]).

Sex-specific differences in seasonal movements have also been observed in bobcats (*Lynx rufus*), with males moving more than females during the dispersal period [69]. In dingoes, we found that subdominant female detections outside of their home range increased slightly compared with males before the breeding season. Subdominant female dingoes may be expected to explore outside their territories to investigate if adjacent areas lack a female, such as occurs in meerkats [70]. Additionally, it could be expected that subdominant female explorations outside of their range would occur before the breeding season, as dispersing females would preferably establish a territory before the breeding season. We found only slight evidence of this, however, and female dispersal may instead be linked to other factors such as resource availability [15].

### 4.2. Dominant pair/single detections

Detections of dominants individually (lone detections) showed limited variation in overall movement patterns by month (figure 3), and their patterns were similar across both territorial zones. This was contrary to what was expected (hypothesis (c)), as dominant individuals in other species preferentially visit home range peripheries during and around the breeding season [41]. While we predicted that the dominant pair would be more likely to be detected together during and immediately preceding the breeding season than at other times, a failure to detect this does not necessarily provide evidence that mate-guarding does not occur at this time. While dominant males may be actively attempting to mate-guard females, the females themselves may actively resist this and spend substantial time alone and on extra-territorial forays [71]. Such failed mate-guarding attempts could result in similar levels of lone dominant detections throughout the year. While noting that the sample size was not large enough to detect a significant change, lone female detections were indeed lowest during the breeding season (around April) (figure 3), and they were almost never detected alone outside their home range, suggesting a level of mate-guarding might occur at this time. Conversely, lone dominant male detections increased outside the home range during the breeding season, but again sample size was too low for this to be considered a significant change.

As extra-territorial forays and copulations are observed in some socially monogamous species [72], we predicted that dominants would expend more resources to guard their territory during the breeding period, as occurs in other canids [41]. We found no evidence that either male or female dominant individuals moved more around the breeding period. Our sampling period may have been too limited (approx. six months each in two years) to detect significant changes in behaviour. Outside the breeding season, dominants may visit their home range periphery enough to scent mark and defend their territories, and maintain this behaviour throughout the pre-breeding season [73]. This may indicate that for dominant individuals, territorial maintenance is a year-round process, as is the case in other species including many canids [27,74].

### 4.3. Dependent offspring affect movement of dominants

In addition to reproductive opportunities beyond the natal territory, extra-territorial forays or increased use of areas within the home range may also be driven by competition within the natal pack (hypothesis (d) [75]. We found that weekly independent dingo detections inside and outside the home range varied by reproductive status and according to the number of pups present in the resident pack (figure 3). For dominant individuals, the proportion of detections within the home range increased with the number of pups from the previous year, suggesting that increased pup care demands may anchor the dominant pair, as occurs in African wild dogs [17]. In Ethiopian wolves, dominants patrolled their territories less with increasing pack size [76]. Whether successful raising of juveniles impacts the number of extra-territorial forays conducted by dominant individuals has not been explored in the literature and requires further study.

Intense competition within a pack can induce dispersal by subdominants [75]. Consequently, we predicted that subdominant dispersal, or pre-dispersal, may be driven by increased resource competition in the natal range [77]. However, although we found that subdominant dingoes were more likely to be detected outside of their home range than dominant individuals were, we found no evidence that this was related to the number of pups in the pack. This result contrasts with meerkats, where subdominants disperse more at both low and high density of competitors and disperse least at an intermediate level [78]. Individuals within the population may be making different decisions on whether to explore or disperse, dependent on mechanisms not identified here. As such, resource availability and the mechanisms behind dispersal events may be more complex than we anticipated [32]. Some studies in other canids suggest benefits to delayed dispersal, regardless of breeding opportunities in the natal pack [79], and these benefits, such as increased safety [30], may help to explain why extra-territorial detections of subdominants were unrelated to proxies for competition within the pack.

Our results highlight the importance of considering individual demography within animal populations when attempting to understand territorial behaviours, rather than treating a population as a homogeneous entity. While we did not find an overall increase in dingo detections around the breeding season, we did see seasonal fluctuations in dingo movement between dingoes in different demographic categories. The time of year, sex, dominance status and the number of pups in the pack all influenced dingo movement in different ways. Future research of dingo behaviour should expand to cover the entire annual cycle and extended longitudinal studies may aid in teasing out the drivers of extra-territorial forays and dispersal in dingoes. Further knowledge of the drivers of movement in carnivores more broadly will aid in targeted management of specific individuals or at specific times of the year.

## Data Availability

Data and relevant code for this research work are stored in GitHub: https://github.com/Brendan-Alting/DingoRangingBehaviour and have been archived within the Zenodo repository [80]. Supplementary material is available online [81].
